# Diabetes prevention and treatment: a global perspective

**DOI:** 10.1007/s00125-025-06511-6

**Published:** 2025-08-12

**Authors:** Christian Herder, Cheryl Pritlove, Nishi Chaturvedi, Hindrik Mulder

**Affiliations:** 1 Institute for Clinical Diabetology, German Diabetes Center, Leibniz Center for Diabetes Research at Heinrich Heine University Düsseldorf, Düsseldorf, Germany; 2 German Center for Diabetes Research (DZD), Partner Düsseldorf, München-Neuherberg, Germany; 3 Department of Endocrinology and Diabetology, Medical Faculty and University Hospital Düsseldorf, Heinrich Heine University Düsseldorf, Düsseldorf, Germany; 4 Li Ka Shing Knowledge Institute, Unity Health Toronto, Toronto, ON Canada; 5 Dalla Lana School of Public Health, University of Toronto, Toronto, ON Canada; 6 Unit for Lifelong Health & Ageing, Institute of Cardiovascular Science, University College London, London, UK; 7 Unit of Molecular Metabolism, Lund University Diabetes Centre, Malmö, Sweden

**Keywords:** Ancestry, Complications, Diabetes, Disparities, Equity, diversity and inclusion, Ethnicity, Geography, Global, Precision medicine

## Introduction

In recent years there has been an increasing focus on precision medicine, including in diabetology [1–3]. However, many studies in this field have two limitations. First, most studies on diabetes prevention and treatment originate from high-income countries (HICs), whereas the burden of diabetes is highest and increasing most rapidly in low- and middle-income countries (LMICs) [4, 5]. Second, and closely related to the first limitation, most studies do not sufficiently capture the global diversity of diabetes aetiology, phenotypes and therapeutic needs based on ancestry, ethnicity and geography [6]. However, there is clear evidence that this diversity is clinically relevant [7, 8]. Currently, global differences in diabetes epidemiology and pathophysiology as well as disparities in diabetes prevention and management are insufficiently understood [4, 8, 9].

Therefore, the overarching aim of the reviews in this special issue of *Diabetologia* is to explore global opportunities and challenges in the prevention and treatment of diabetes, addressing topics from global differences in the prevalence of diabetes types (e.g. type 2 diabetes, hyperglycaemia in pregnancy and monogenic diabetes) to the development of complications, challenges of diabetes prevention in different populations (e.g. migrant populations and the Arctic Inuit), disparities in access to diabetes technologies and therapeutics, and the relevance of ancestral and/or ethnic diversity in precision medicine. In addition, we present a review on diabetes management in the unique context of man-made and natural disasters, and the EASD Global Council looks to the future, calling for a coordinated global approach to diabetes research and care to promote equitable healthcare for all.

The articles featured in this special issue situate *Diabetologia* as a leading forum for high-quality research that engages critically with these global challenges, advancing our understanding and catalysing meaningful responses to the complex, interconnected drivers of diabetes worldwide. Collectively, these articles enable rigorous evaluation and timely communication of important findings, including explanations for population differences in causes and consequences of diabetes, and informing evidence-based strategies for effective prevention and management. Additionally, this collection of articles underscores the importance of addressing the diversity of diabetes phenotypes and the geopolitical and socioeconomic challenges to prevention and treatment faced by LMICs if any global progress is to be made.

## Global opportunities and challenges

### Race, ethnicity and ancestry

To meaningfully advance prevention, care and outcomes worldwide, diabetes research must better reflect the diversity of global populations and the complex, intersecting factors that drive disease. In their review, Chaturvedi at al [10] argue that we must seek explanations for population differences in diabetes risk and outcomes, which are shaped by a dynamic interplay of genetic, environmental and structural influences, many of which are modifiable. While documenting group-level differences remains important, doing so without examining the multifaceted and intersecting root causes risks reinforcing simplistic or stigmatising narratives that obscure the broader determinants of health. The authors therefore urge researchers to approach population categorisation thoughtfully, selecting and clearly defining terms such as ‘race’, ‘ethnicity’ and ‘ancestry’ with care, transparency and attention to who is assigning these terms. An interdisciplinary approach that bridges biomedical and social science, and meaningfully includes the voices and experiences of those most affected, is needed to generate more accurate, equitable and globally relevant knowledge and interventions.

### Type 2 diabetes

Diabetes prevalence has increased rapidly in recent decades, with more than one in nine people (11.1%) worldwide estimated to be living with the disease. In their review, Gong et al [11] discuss how type 2 diabetes prevalence varies considerably by country and region and is impacted by shifting population demographics, changes in incidence and mortality, variations in diabetes diagnosis, treatment and prognosis, and epidemiological and nutritional transitions. The authors explore the various drivers of these differences, including urbanisation and industrialisation, genetic predisposition, environmental factors, migration and disparities in healthcare access and diagnostic practices. Understanding these drivers may reveal important ways to identify those most at risk, helping to design prevention strategies suited for the diversity observed in diabetes prevalence.

### Hyperglycaemia in pregnancy

Similarly, the global prevalence of hyperglycaemia in pregnancy, comprising known pre-existing diabetes and diabetes diagnosed during pregnancy, including gestational diabetes, is also increasing. In their review, Yuen et al [12] discuss how the likelihood of being diagnosed with diabetes before or during pregnancy depends on ethnic background and geographical location. These differences in diagnosis could be due to social and economic factors either directly impacting biology or influencing health policy, access to healthcare and economic stability. Geography can also exert unique effects due to seasonality, pollution and altitude. The authors conclude that there is a need for a standardised and simple approach to diagnosing diabetes in pregnancy, to help provide a clear understanding of these effects and enable any differences to be eliminated.

### Monogenic diabetes

Russ-Silsby et al [13] go on to discuss monogenic forms of diabetes, which are responsible for a small but important subset of diabetes cases worldwide. Focusing on the two most common subtypes, MODY and neonatal diabetes, they describe how advances in precision medicine and genomic technologies have led to the discovery of new disease genes and improved treatments. The authors also examine some of the key challenges currently facing the field, including issues with variant interpretation stemming from variable disease penetrance, the possibility of missed genetic diagnoses depending on the testing strategies used, and inequitable diagnosis and treatment of monogenic diabetes due to genetic ancestry bias and inequitable access to genetic testing. The authors conclude by highlighting future directions in diagnosing and treating monogenic diabetes.

### Metabolic disorders in young people

Importantly, obesity and diabetes, including type 2 diabetes, are becoming increasingly common in children and teenagers around the world and therefore research into these groups is vital. In their review, Boddu et al [14] discuss how these metabolic conditions in young people can lead to serious health problems later in life, such as heart disease and early death. Poor diet, lack of physical activity, and other lifestyle and environmental factors increase the risk of metabolic disorders, especially when combined with a family history of these conditions. The authors highlight that young people diagnosed with metabolic conditions often face more severe and earlier health issues than those diagnosed in adulthood, which is especially concerning in LMICs, where access to healthcare and treatment may be limited. Promoting healthy habits, improving access to healthcare and focusing on prevention, especially in underserved communities, are needed to improve long-term outcomes and support the well-being of children and adolescents.

### Complications of diabetes

Addressing diabetes-related complications is key to effective diabetes management and reducing the risk of serious long-term issues. In their review, Luk et al [15] discuss how the development of complications in people with diabetes is shaped by complex interactions between biological, clinical and social factors that vary across regions and ethnicities. Certain high-risk ethnic groups have distinct biological profiles that contribute to both an earlier onset of diabetes and more rapid progression of organ damage. The authors conclude that social determinants of health, such as socioeconomic status, cultural practices, health literacy and access to healthcare, are likely to be more important than biological factors in explaining the observed differences. Globalisation and the emergence of medical innovations may further widen these disparities over time.

### Diabetes in migrant populations

Three in four adults with type 2 diabetes live in LMICs, and migrant populations from LMICs residing in HICs are impacted more severely by type 2 diabetes and related complications than their host populations, posing a significant public health challenge. In their review, Bennet and Agyemang [16] discuss how this increased risk is related to lifestyle changes associated with migration, health literacy or socioeconomic barriers hindering access to healthcare. Among those already diagnosed with type 2 diabetes, suboptimal care and poor management contribute to their high risk of diabetes-related complications and mortality. The authors highlight that, in the context of international migration from LMICs to HICs, prevention and standard care are often not sufficient. Culturally tailored and gender-sensitive strategies that address social determinants of health and involve affected populations, stakeholders and communities are essential to mitigate the burden of type 2 diabetes in migrant populations. The authors conclude that future research should focus on evaluating the long-term effectiveness of these strategies and creating successful models for broader implementation.

### Diabetes in Inuit populations

From being almost non-existent in the 1960s, diabetes is now an important health concern among Inuit populations across Alaska, Canada and Greenland. In their review, Nielsen et al [17] explore how diabetes affects the Inuit, who share genetic ancestry but live within distinct healthcare and social systems, which are challenged by remote locations, limited healthcare infrastructure and frequent staff turnover. While reported estimates of diabetes and related comorbidities prevalence vary, these differences are likely to reflect inconsistent screening and data collection rather than being true disparities in disease burden. The authors highlight how unique genetic variants found only in Inuit populations may increase diabetes risk but also facilitate more targeted, personalised care. They also describe how diabetes care is delivered across regions, highlighting persistent barriers as well as different approaches to culturally adapted strategies such as traditional food programmes. Central to progress is ensuring that Inuit communities retain ownership of their health data, enabling trust, and implementing community-led research and care.

### Diabetes technologies, therapeutics and disparities

Recent technological and therapeutic advances in the management of type 1 diabetes (e.g. automated insulin delivery) and type 2 diabetes (e.g. sodium‒glucose co-transporter 2 inhibitors and glucagon-like peptide-1 receptor agonists) have improved both glycaemic outcomes and quality of life for individuals living with diabetes. However, studies have consistently demonstrated disparities in access to diabetes technologies and therapeutics in minoritised groups. In their review, Addala et al [18] apply neo-materialist theory to dissect how structural and systemic inequities shape disparities in four key domains of diabetes care: clinical trial participation, use of diabetes technology, immunotherapies and digital tools. The review highlights the under-representation of minoritised groups in trials and disparities in use of emerging treatments, driven by systemic barriers such as exclusionary design, lack of culturally grounded engagement and inequitable access. Despite progress, use of continuous glucose monitors and automated insulin delivery systems remains uneven across ethnic, socioeconomic and insurance lines. Gaps in digital infrastructure further exacerbate these divides, particularly in telemedicine access. The authors call for structural reforms—from inclusive research designs to digital equity initiatives—to ensure that the benefits of diabetes innovation are equitably distributed.

### Ethnic diversity in precision medicine

People from different ethnic backgrounds present with diabetes in distinct ways, yet most current treatments do not reflect this diversity. In her review, Shivani Misra [19] explores how precision medicine can help tailor care to individual needs, focusing on the importance of including ethnically diverse populations in research, so that new diagnostic tools and treatments work for everyone. The review examines how genetics, lifestyle, environment and access to healthcare all contribute to differences in disease progression and response to treatment. It highlights the pitfalls of under-representation of ethnic groups in research and presents practical solutions for developing precision care for different ethnic groups and in all-resource settings. By combining both personalised and population-level strategies, the review sets out a path towards fairer and better diabetes care.

### Diabetes and disasters

Natural and man-made disasters present particular challenges for people with diabetes. With both the global prevalence of diabetes and the frequency of natural and man-made disasters increasing, it is inevitable that many people with diabetes will be impacted by these life- and health-threatening catastrophic events. Continued access to diabetes care and medicines, in particular insulin, is essential and a basic human right, but such resources are often limited during disasters, particularly in less advantaged or less well-prepared regions. In their review, Boulton and Jenkins et al [20] share their diabetes-related experiences of disasters and review the consequences of and how to prepare for and respond to disasters. Recent examples of the COVID-19 pandemic in India, the war in Ukraine and the war/blockade in the Tigray region of Ethiopia are described, and the importance of prevention, preparedness, response and recovery for people with diabetes, healthcare professionals and diabetes associations and governments are discussed. The authors conclude that post-disaster assessments and sharing of experiences are crucial to alleviate the risks of morbidity and mortality for people with diabetes in disaster-impacted regions across the world.

### Global challenges

As the reviews in this special issue have highlighted, diabetes is a rapidly growing global health challenge, especially in LMICs. In the final review, the EASD Global Council emphasises the urgent need to improve both diabetes research and care worldwide. Giorgino et al [21] explore critical gaps in access to medications such as insulin, the under-representation of vulnerable populations in research, and the difficulty of applying new scientific discoveries, such as digital tools and personalised medicine, in everyday healthcare settings. The authors also emphasise the importance of equitable education and training for healthcare providers and call for the development of international standards for diabetes care, such as global screening programmes and universal care checklists. By identifying major challenges and offering practical solutions, from improving data systems to strengthening global collaboration, the review provides a roadmap for making diabetes care more inclusive, effective and resilient to future health threats.

### Lived experience

We have also included the diverse lived experiences of individuals with diabetes across the globe in this special issue. In their letter, Mytkolli et al [22] highlight how geographical location, income and disasters can impact access to insulin, devices and supplies; the crucial roles that family, friends and peers play in supporting those with diabetes; how the expertise of people living with diabetes should be harnessed to boost innovation; and the resilience shown by people with diabetes in managing their condition. Representing these varied experiences gives us a more complete and inclusive understanding of the opportunities and challenges, moving beyond generalised statistics and recognising the impact of diabetes on individuals from different backgrounds

## Future directions

Taken together, the reviews in this special issue illustrate the diversity of different diabetes types across the world, the impact of ancestry, ethnicity and geography on diabetes prevalence and risk, and the close interplay between the aforementioned factors and other determinants of health. The reviews also point towards the remaining challenges. It is important to note that there is a lack of consensus around definitions and terminologies when referring to population subgroups, which explains why a uniform terminology across the articles in this special issue is lacking, particularly when referring to older literature.

*Diabetologia,* as a leading journal in the diabetes field, intends to serve as a natural home for research that explores the intricate relationships between the multiple factors influencing diabetes, with a global reach and ambition. As such, recent articles in the journal have addressed the diversity in diabetes risk related to sex and gender [23, 24], socioeconomic factors and equity [25–27], and environmental factors associated with climate change [28]. As a journal, we are deeply committed to advancing research that foregrounds equity, diversity and inclusion in the understanding and management of diabetes and its complications. This special issue embodies our belief that improving diabetes care and outcomes requires a comprehensive approach, one that considers the complex interplay of biological, social, cultural, economic, geographical and political determinants of health at both individual and population levels. Our ultimate goal is to generate knowledge that empowers people living with diabetes, healthcare providers, policymakers, global health leaders and other stakeholders in their actions to prevent and manage disease. These efforts must be global in scope and inclusive of all communities, ensuring that advances in diabetes prevention and care are accessible to everyone, unhindered by geographical borders and social and economic barriers.

## Abbreviations

HICs: High-income countries
LMICs: Low- and middle-income countries
